# SynD: Australian synthetic health data community of practice

**DOI:** 10.23889/ijpds.v9i5.2766

**Published:** 2024-09-18

**Authors:** Ben Hachey, Jodie Austin, James Boyd, Teyl Engstrom, Amir Marashi, Clare Morgan, Reji Philip, Keren Pointon, Meagan Snewin, Clair Sullivan, Melanie Haines

**Affiliations:** 1 University of Sydney; 2 University of Queensland; 3 La Trobe University; 4 Digital Health CRC

## Objectives

The current workflow for health data research in Australia is inefficient. After funding is secured, researchers often face delays of months or years to access the necessary data. Synthetic data could significantly improve the pace and impact of health data research but lacks foundational infrastructure. We aim to develop this infrastructure and support the use of synthetic data to improve data access and research quality across Australia.

## Approach

We held two workshops with Australian groups working on synthetic data. The format included participant updates and invited talks on international approaches to synthetic data and health data research. Workshops collected use cases and stimulated discussion on national collaboration. A facilitator then led thematic analysis to draft a consensus roadmap and terms of reference towards national synthetic data infrastructure.

## Results

We recruited 18 participants. Participants were cross sectoral: universities (9), research funding bodies (5), state health departments (4). Represented six states and territories: Queensland (6), New South Wales (3), Victoria (3), Western Australia (3), Australian Capital Territory (2), South Australia (1). Gender: women (11), men (7). The roadmap includes stakeholder engagement, a governance framework, and training events.

## Conclusion

SynD is an Australian community of practice for synthetic health data. Our mission is to unlock the value of health information through synthetic data to advance research, education, innovation and service delivery within the health and care sector. This collaborative effort should ensure a harmonised approach to the safe and effective utilisation of synthetic data to enhance health outcomes across Australia.

